# Characterizing cyclin-dependent kinase 12(CDK12)-altered aggressive prostate cancer: a twelve-case series

**DOI:** 10.1007/s10147-022-02248-z

**Published:** 2022-10-21

**Authors:** Tomohiro Iwasawa, Takeo Kosaka, Yota Yasumizu, Hiroshi Hongo, Yoshinori Yanai, Yuto Baba, Kazuhiro Matsumoto, Kohei Nakamura, Hiroshi Nishihara, Mototsugu Oya

**Affiliations:** 1 Department of Urology, Keio University School of Medicine, 35 Shinanomachi, Shinjuku-ku, Tokyo, 160-8582 Japan; 2 Genomics Unit, Keio Cancer Center, Keio University School of Medicine, 35 Shinanomachi, Shinjuku-ku, Tokyo, 160-8582 Japan

**Keywords:** Cyclin-dependent kinase 12 (CDK12), Prostate cancer, Genomic analysis, Precision oncology, Case series

## Abstract

**Background:**

Prostate cancer harboring cyclin-dependent kinase 12 (CDK12) abnormalities is a hot topic due to its distinctive clinical features, such as sensitivity to immune checkpoint inhibitors. In the last few years, precision medicine using comprehensive genome sequencing has become familiar, and the era of precision oncology has arrived in the field of prostate cancer. This study aimed to present the demographic characteristics of patients with *CDK12* alterations.

**Methods:**

In 12 patients with detected *CDK12* alterations in our hospital between 2015 and 2021, we evaluated their genomic features and clinical course. *CDK12* allelic status was classified into three groups: monoallelic loss, potentially biallelic loss, and biallelic loss based on the genome analyses.

**Results:**

Seven patients already had metastatic cancer at the time of diagnosis, and all 12 patients had Gleason grade  ≥ 4. Most cases of biallelic loss or potentially biallelic loss were metastatic cancers at the initial staging, and all these cases were categorized into Gleason grade 5. Two of the 12 patients had *BRCA2*/*RB1* co-loss, and the other two had whole genome duplication. Five patients had a long-term survival of  > 6 years, but two patients died within 4 years of diagnosis.

**Conclusion:**

This is the first Japanese prostate cancer case series with *CDK12* alterations. *CDK12*-altered prostate cancer is a heterogeneous disease, and accumulating cases with detailed information leads to precision oncology.

**Supplementary Information:**

The online version contains supplementary material available at 10.1007/s10147-022-02248-z.

## Introduction

Recently, precision medicine using comprehensive genome sequencing has become popular, and the era of precision oncology has arrived in the field of prostate cancer. A typical example of precision oncology for prostate cancer is the use of poly (adenosine diphosphate ribose) polymerase (PARP) inhibitors for patients with DNA-damage response defects. Cyclin-dependent kinase 12 (CDK12) is one of the frequently mutated genes in prostate cancer [1] and sometimes considered one of the DNA-damage response genes [2]. In a clinical trial evaluating the efficacy of a PARP inhibitor in castration-resistant prostate cancer (CRPC), a subgroup analysis of the patients with *CDK12* mutation did not show survival advantage [3].

A study in 2018 reported that immune checkpoint inhibitors (ICIs) might be efficient for prostate cancer with biallelic *CDK12* loss [4]. Since then, there has been a growing number of studies on *CDK12*-altered prostate cancer, with most reporting that patients with this type of cancer have poor prognosis [5–7]. However, it remains unclear which treatment is truly effective for *CDK12*-altered prostate cancer or whether *CDK12* monoallelic loss can be treated in the same manner as biallelic loss.

We have treated 12 cases of *CDK12*-altered prostate cancer from various backgrounds, including monoallelic and biallelic loss and localized and metastatic cancer. This study aimed to present the demographic characteristics of patients with *CDK12* alterations.

### Patients and methods

Of the patients with prostate cancer who underwent genomic testing at Keio University Hospital (Tokyo, Japan) between 2015 and 2021, 12 had possibly deleterious *CDK12* gene alterations. Written informed consent was obtained from all patients under the approval of the Ethics Committee of our hospital.

Tissues used for genomic testing included prostate needle biopsy specimens at the time of diagnosis, radical prostatectomy specimens, prostate rebiopsy specimens at the time of progression, and specimens of metastatic sites. The modalities of genomic testing included a cancer-related gene panel test using tumor tissue (PleSSision-Rapid^®^) or circulating cell-free DNA (cfDNA) (FoundationOne^®^ Liquid CDx) and whole exome sequencing (WES) using tumor tissue (PleSSision-Exome^®^). The cutoff value of variant allele frequency (VAF) was 4% and mutations were called when the minimum gene coverage was  > 20 reads and the minimum variant coverage was  > 2 reads for PleSSision-Rapid^®^ or PleSSision-Exome^®^. For FoundationOne^®^ Liquid CDx, the detailed criterion for variant calling is not disclosed, but the reported variants were considered as significant despite the VAF being low.

The copy number of each gene was calculated as the median value of all sequencing reads covering the target genes and compared with the median value of the control samples. The estimated copy number (eCN) of the tumor cells was calculated using the following formula: eCN = 2 + {(measured copy number−2) /proportion of tumor cells}. In the algorithm we used, the allosomal copy number reference value was also set to 2, the same as that for the autosomes, for computational convenience. When referring to copy number alterations, we defined eCN ≥ 3 as “gain,” eCN ≥ 4 as “amplification,” and eCN ≤ 1 as “loss.” Homozygous deletion was determined if the eCN was nearly 0. If a case had “major allele ploidy  > 1.5 (eCN > 3) on at least 50% of at least 11 autosomes” [8], the case was judged as whole genome duplication (WGD). All cfDNA samples and tumor samples with tumor content  < 30% were excluded from copy number analysis.

High tumor mutation burden (TMB) was defined as a mutation rate  > 10 SNVs/Mbp. Microsatellite status was evaluated using MSIsensor in PleSSision-Exome^®^, and high microsatellite instability (MSI) was defined as  ≥ 20%. For PleSSision Rapid, microsatellite status was examined based on a panel test for five MSI loci. FoundationOne^®^ Liquid CDx analyzed 1765 loci but only showed whether the case had high of low MSI.

Cases with monoallelic loss were defined as those having a deleterious mutation in *CDK12*, and the definition of cases with biallelic loss was similar to that of “loss of function alterations” used by Sokol et al. [9]: (a) mutations with loss of heterozygosity at the wild-type allele, (b) copy number loss (homozygous deletion), or (c) two or more *CDK12* genomic alterations in each sample. For short variants, those registered in the database as pathogenic were considered deleterious mutations and those with computationally predicted damage alone were considered as variants of uncertain significance (VUS). We defined those cases with one allele of pathogenic mutation and the other of VUS as having potentially biallelic loss. The definition of CRPC was adopted from the Prostate Cancer Working Group 3 [10]. To determine the treatment efficacy, a PSA50 decline was defined as  ≥ 50% decrease in prostate-specific antigen (PSA) levels from baseline, while a PSA30 decline was defined as  ≥ 30% decrease.

## Results

### Patient demographics

For 12 patients with *CDK12*-altered prostate cancer, the median age at initial diagnosis was 67 years, the median PSA level was 18.5 ng/dL, and all patients had a Gleason grade  ≥ 4. Five patients had localized disease, and seven had metastases at the time of diagnosis. The specimens used for genomic testing were pretreatment prostate biopsies in three cases, prostatectomy specimens in two cases, biopsy after CRPC samples in five cases, and cfDNA in two cases. The tumor content of each tissue sample ranged from 20 to 80% (median, 40%). The modalities of the genomic testing were comprehensive cancer-related gene panel test for seven cases (including two cases using cfDNA) and WES for five cases (Table 1).

**Table 1:** Patient demographics

	All patients *n* = 12		Monoallelic loss (including copy number not acquired cases) *n* = 4		Potentially biallelic loss *n* = 3		Biallelic loss *n* = 5	
Age at diagnosis, median (range), years	67	(54– 81)	63	(54–63)	60	(54–63)	72	(56–81)
Initial stage, *n* (%)								
Localized	5	(42)	3	(75)	1	(33)	1	(20)
Metastatic	7	(58)	1	(25)	2	(67)	4	(80)
Genetic testing, *n* (%)								
Panel test	7	(58)	3	(75)	1	(33)	3	(60)
WES	5	(42)	1	(25)	2	(67)	2	(40)
Initial PSA, median (range), ng/mL	18.5	(3.9–643)	5.8	(4.1–18.3)	370	(18.7–439)	36.8	(3.9–643)
Gleason grade group, *n* (%)								
4	2	(17)	2	(50)	0	(0)	0	(0)
5	10	(83)	2	(50)	3	(100)	5	(100)
Initial HNPC therapy, *n* (%)								
ADT or CAB	9	(75)	3	(100)	3	(100)	2	(40)
Upfront abiraterone	2	(17)	0	(0)	0	(0)	2	(40)
Platinum-based therapy	1	(8)	0	(0)	0	(0)	1	(20)
Duration of initial hormonal therapy, median (range), months	26	(7–108)	32	(7- 48)	45	(9–108)	22	(22–24)
First-line CRPC therapy, *n* (%)								
Abiraterone	2	(29)	0	(0)	1	(33)	1	(50)
Enzalutamide	3	(42)	1	(50)	1	(33)	1	(50)
Docetaxel	2	(29)	1	(50)	1	(33)	0	(0)

*WES* whole exome sequencing, *PSA* prostate-specific antigen, *HNPC* hormone-naïve prostate cancer, *ADT* androgen deprivation therapy, *CAB* combined androgen blockade, *CRPC* castration-resistant prostate cancer

### Genomic findings

The mean coverage depth of tissue specimen analyses ranged from 189.0 to 579.0 (median, 482.9). Of the two cases of cfDNA panel testing, one had a tumor genome fraction of 11%, and the other had “elevated tumor genome fraction not detected.” The latter case indicates the possibility of lower amount of circulating tumor cfDNA but does not compromise the reliability of any reported alterations. The coverage depth and copy numbers for these two cases were unavailable. Two of the 12 cases had a mutation rate  > 10 SNVs/Mbp, which showed high TMB, but none had high MSI (Tables 2, 3).

**Table 2:** Genome characteristics

ID	Genomic testing	Sample	Mean depth (reads)	Tumor content (%)	TMB (SNVs/Mbp)	MSI (%)
KOURO_31–408	WES	Prostate biopsy	203.9	40	2.0	9.66
KOURO_20–298	WES	Lymph node biopsy	723.9	70	3.7	4.86
KOURO_20–331	WES	Prostate biopsy	482.9	20	2.3	0.02
KOURO_20–334	WES	Prostate biopsy	189.0	80	20.7	5.98
KOURO_20–346	WES	Prostate biopsy	579.0	30	3.4	0.21
KOURO_19–598	Panel	Lung metastasectomy	487.4	30	4.0	Stable
KOURO_7–15	Panel	Prostatectomy	393.6	50	3.5	Stable
KOURO_7–24	Panel	Prostatectomy	292.0	50	7.0	Stable
KOURO_1–6	Panel	Rectum biopsy	516.4	40	12.8	Stable
KOURO_2574	Panel	Prostate TUR	530.2	40	6.7	Stable
KOURO_liq-11002	Panel (cfDNA)	Blood liquid biopsy	N/A	11	1.0	Stable
KOURO_liq-11703	Panel (cfDNA)	Blood liquid biopsy	N/A	Not detected	4.0	Stable

*TMB* tumor mutation burden, *SNV* single nucleotide variant, *MSI* microsatellite instability, *WES* whole exome sequencing, *TUR* transurethral resection, *cfDNA* cell-free DNA, *N/A* not available

**Table 3:** Genome characteristics of *CDK12*

ID	*CDK12* allele 1		*CDK12* allele 2		*CDK12* copy number	*CDK12* status
	Mutation	VAF (%)	Mutation	VAF (%)		
KOURO_31–408	H467Tfs*19	28.1	I730del (VUS)	60.4	Gain (CN = 3)	Potentially biallelic loss
KOURO_20–298	G101Dfs*23	37.5	C952R (VUS)	32.5	Neutral	Potentially biallelic loss
KOURO_20–331	T1463Nfs*50	7.4	N/A		Neutral	Copy number not acquired
KOURO_20–334	P1005-S1006 del	68.9	N/A		Gain (CN = 3)	Biallelic loss (Mutation with LOH)
KOURO_20–346	S1044Lfs*13	24.4	N/A		Neutral	Biallelic loss (Mutation with LOH)
KOURO_19–598	Q937*	72.2	R1008Q (VUS)	25.5	Gain (CN = 3)	Potentially biallelic loss
KOURO_7–15	S30Tfs*26	29.6	D962Mfs*11	18.9	Neutral	Biallelic loss (Pathogenic × 2)
KOURO_7–24	L908R	15.1	N/A		Neutral	Monoallelic loss
KOURO_1–6	E461*	20.6	N/A		Neutral	Monoallelic loss
KOURO_2574	W719fs*14	6	c.2768 + 1 G > A†	15.1	Neutral	Biallelic loss (Pathogenic × 2)
KOURO_liq-11002	E173fs*18	7.8	K756R	15.4	N/A	Biallelic loss (Pathogenic × 2)
KOURO_liq-11703	c.2610-1G > A†	0.2	N/A		N/A	Copy number not acquired

*VAF* variant allele frequency, *VUS* variant of unknown significance, *CN* copy number, *N/A* not available

†splice-site variant

**Fig. 1:**
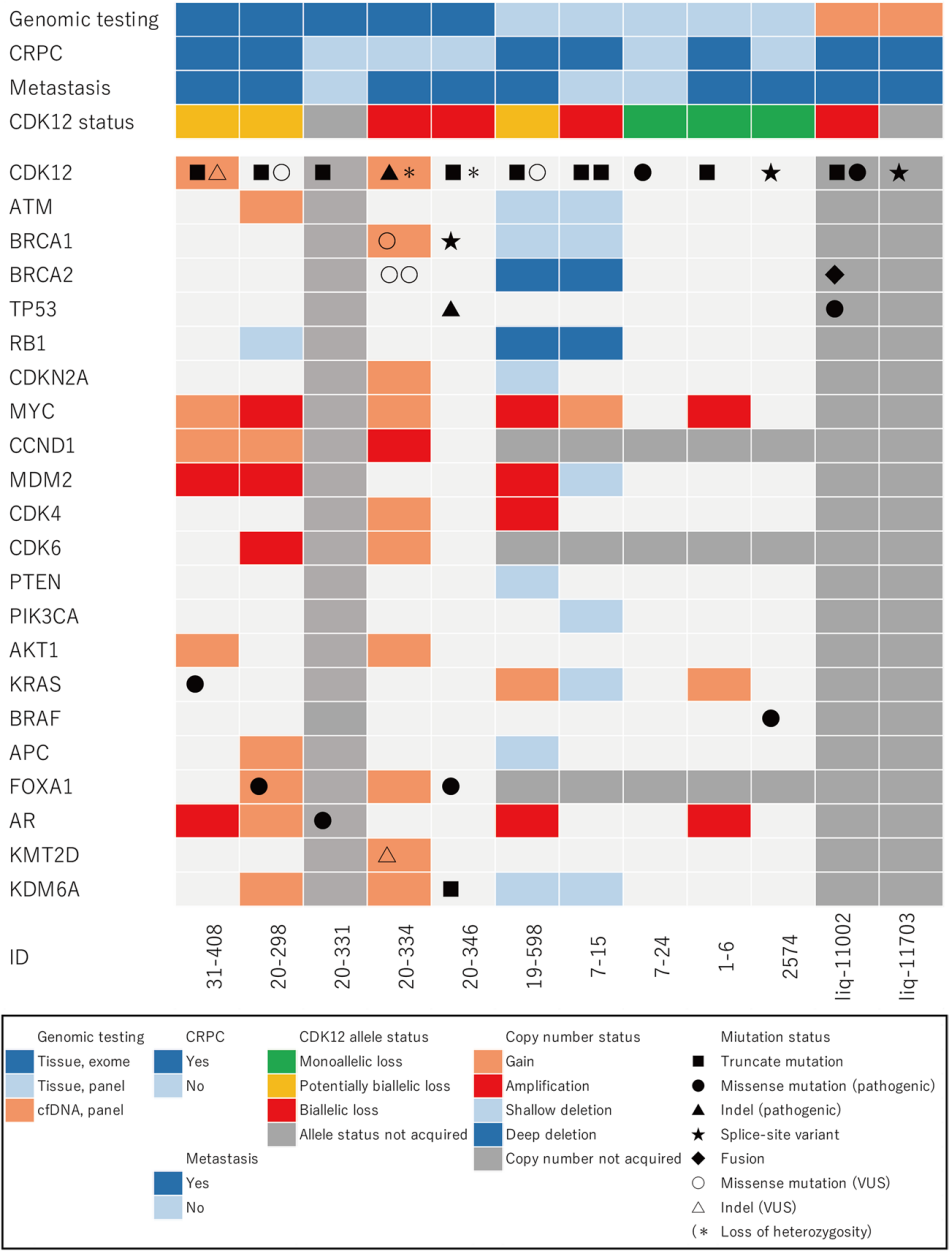
Genomic landscape of patients with *CDK12* alteration “Yes” and “No” for CRPC and metastasis indicate the status of the case at the time of sample collection for genome analysis. *CRPC* castration-resistant prostate cancer, *VUS* variant of unknown significance

Four of the 12 cases had *CDK12* monoallelic loss: one with a missense mutation, one with a splice site variant, and two with a truncating mutation. Three cases with both a truncating mutation and short variant that were computationally predicted as damaging but not registered in the genome database were classified into the potentially biallelic loss group. Of the five cases of biallelic loss, none had homozygous deletion, two had a mutation with loss of heterozygosity, and three had two or more pathogenic mutations within a sample. For the two cases of mutation with loss of heterozygosity, although there was no decrease in CDK12 copy numbers in both of them, we judged them to be biallelic loss because only the pathogenic allele was present as the VAF was almost equal to the tumor content (Fig. 1).

**Fig. 2:**
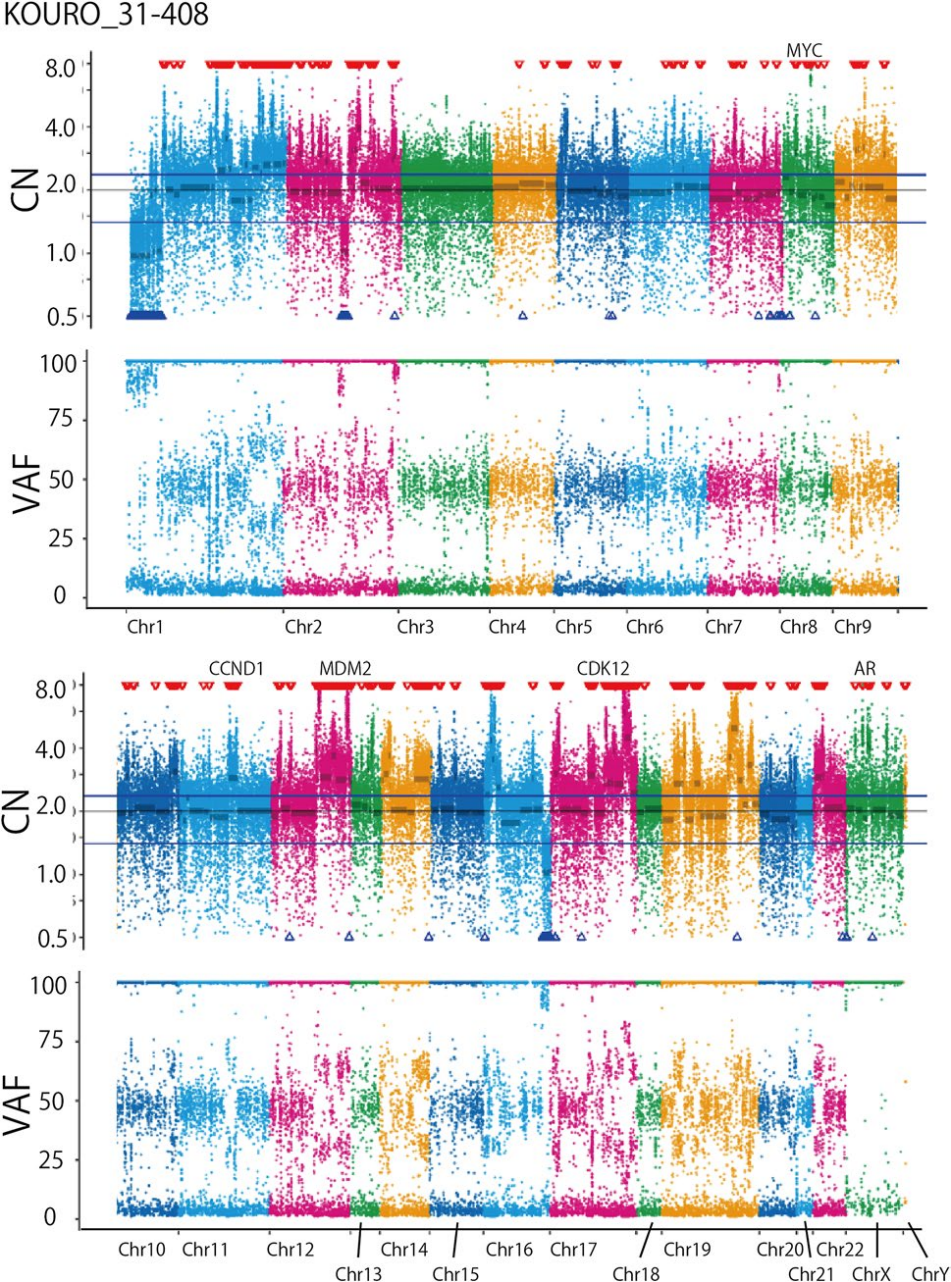
Copy number plot of representative case (KOURO_31-408). Vertical axis shows the row copy number before correction for tumor content. The three horizontal lines in the center represent the first quartile, the median, and the third quartile value for the copy number of whole chromosomes. The red and blue triangles indicate the region of copy number gain or copy number loss, respectively. *CN* copy number, *VAF* variant allele frequency, *Chr* chromosome

Coexisting genomic features included androgen receptor (*AR*) amplification in three cases and *Myc* amplification (eCN > 4) in three cases. *BRCA2* and *RB1* concurrent homozygous deletion was found in two cases (KOURO_19-598 and KOURO_7-15), and these cases presented copy number disturbance characteristic of homologous recombination deficiency (HRD). Two other cases (KOURO_20-334 and KOURO_20-298) apparently had WGD, and one case (KOURO_31-408) presented a characteristic copy number plot with numerous small copy number gains (Fig. 2, Figure S1).

### Treatment response

**Fig. 3:**
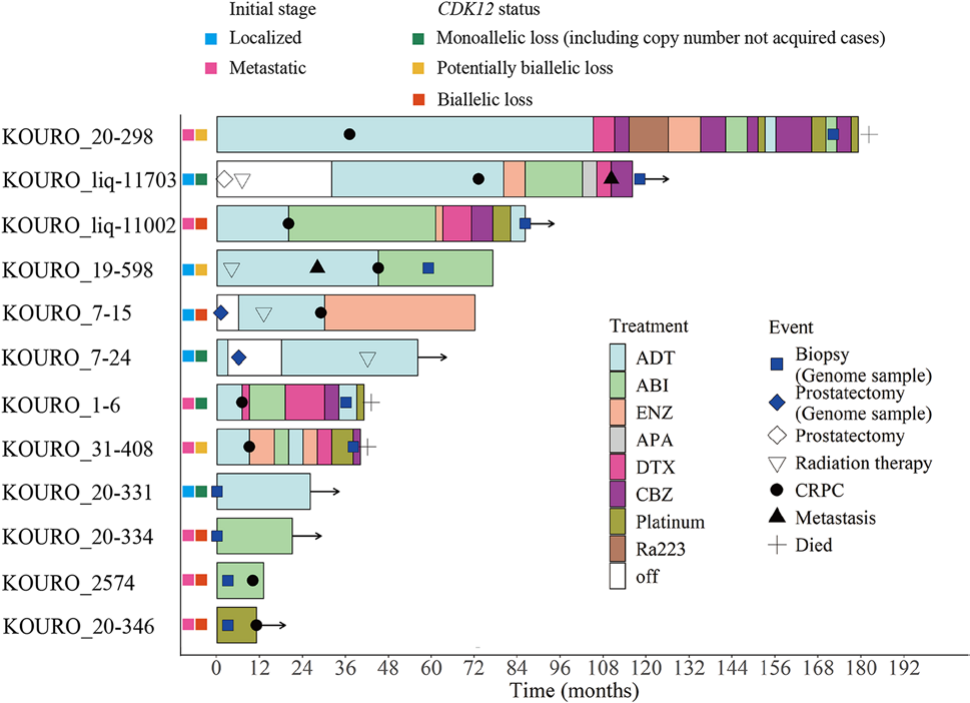
Swimmer plot of patients with CDK 12 alteration. Arrows indicate survival or ongoing treatment. *ADT* androgen deprivation therapy (including combined androgen blockade), *ABI* abiraterone, *ENZ* enzalutamide, *APA* apalutamide, *DTX* docetaxel, *CBZ* cabazitaxel, *Ra223* radium-223; *CRPC* castration-resistant prostate cancer

For first-line CRPC therapy, enzalutamide was administered to three patients, abiraterone to two patients, and docetaxel to two patients. PSA50 decline for first-line CRPC therapy was achieved in all patients using abiraterone or enzalutamide. However, only a few patients achieved PSA50 decline with any subsequent CRPC therapy (Table S2, Fig. 3).

## Discussion

We present the first case series of 12 Japanese patients with *CDK12*-altered prostate cancer from various backgrounds. Five patients had long-term survival of  > 6 years, but two patients died within 4 years of the diagnosis. Some of the genome analyses were performed for research purposes using samples obtained at the time of diagnosis or prostatectomy, and the others were performed after CRPC to find available treatment in clinical practice. Although it is undeniable that CDK12 alterations may have therapeutic causes, a study comparing the primary lesion and metastasis reported CDK12 alterations to be an early event [1]. Therefore, we did not exclude post-treatment samples from our study.

KOURO_31-408, a case of metastatic prostate cancer with an aggressive course, showed a copy number plot similar to that previously reported as typical of *CDK12* biallelic loss [4], and the small copy number gains might reflect focal tandem duplications (FTDs). This patient had a truncate mutation and an in-frame *CDK12* deletion, and the latter was judged as a variant of unknown significance by the database but possibly pathogenic by computational prediction.

KOURO_19-598 and KOURO_7-15 had similar genomic characteristics and achieved long-term survival. The two cases had *BRCA2*/*RB1* co-loss, which was previously reported to have a poor prognosis [11], in addition to *CDK12* alteration. The copy number plots in these cases presented a plot of copy number perturbations as shown in HRD. KOURO_19-598 was a case of metastasectomy for oligoprogression during abiraterone therapy [12]. KOURO_7-15 underwent long-term enzalutamide therapy for biochemical recurrence after radical prostatectomy and salvage radiation therapy. The similarity between these two cases is that they underwent multimodal therapy, while the tumor burden was low.

A question in these patients is whether the driver event is *BRCA2*/*RB1* co-loss or *CDK12* biallelic loss. On one hand, HRDs lead to genomic instability and may cause passenger mutations in *CDK12*. Although TMBs were statistically high in cases with *BRCA1/2* mutations, not all of these cases had high TMB, and more than half of these cases had normal TMB [13]. Given that the TMB of our cases were 4.0 and 3.5 mut/Mbp, respectively, neither of which had high TMB, *CDK12* biallelic mutation seems to show a coincidence with our case of low TMB. On the other hand, it is uncertain whether *CDK12* biallelic loss may cause the co-loss of *BRCA2*/*RB1* because FTD due to *CDK12* biallelic loss usually shows copy number change with a size of 10 kb to10 Mb and a large copy number change across multiple genes is not typical. There is no doubt that these two cases have genomic instability, but we could not reveal which genomic event preceded.

WGD, which is a copy number gain of the entire chromosomes, was noted in two cases of metastatic prostate cancer. One of them (KOURO_20-298) was a case with multiple bone and lymph node metastases at the time of diagnosis that received hormonal therapy and chemotherapy for a long period [14]. The case underwent genomic testing after multiple treatments, and it is unknown when the *CDK12* alteration occurred. In the other case (KOURO_20-334), WGD was detected in the pretreatment sample. This case also had a high TMB, which might be a measurement error in terms of DNA quality. *CDK12* alterations may cause an increase in the copy number in a small region, and the copy number plot formed by FTD alone usually exhibits a bumpy shape with a baseline of copy number of 2. From the aspect of the copy number plot’s shape, it is unlikely that the accumulation of FTD has led to WGD in these cases. Adding the whole genome sequence or RNA sequence would provide more accurate information; however, we did not perform these analyses.

We classified the *CDK12* status into three groups (monoallelic loss, potentially biallelic loss, and biallelic loss) and compared their characteristics. Most cases of biallelic loss or potentially biallelic loss were metastatic cancers at the initial stage, and all these cases had Gleason grade 5. Of the three cases of monoallelic loss, only one case (KOURO_1-6) had an aggressive course, and the remaining two cases had localized cancer and an indolent course. KOURO_1-6 had no remarkable genomic features other than *MYC* amplification, and we could not find the reason for the rapid clinical course from genomic testing.

As several studies have demonstrated that copy number of specific regions frequently increased in prostate cancer with *CDK12* alteration [4, 15], we examined the copy numbers of representative gene loci with *CDK12* alterations. Copy number gains of *CCND1* and *MDM2* were observed in three of four cases and three of nine cases, respectively. We examined the frequencies of CCND1 and MDM2 copy number gains in cases with CDK12 mutations in the public data on advanced prostate cancer [16] and found these to be 50% and 23%, respectively, which is generally consistent with our data. Because these genes frequently have copy number gain in metastatic prostate cancer, it is uncertain whether the copy number gains of our small cohort were associated with *CDK12* alterations.

Next, we attempted to examine the therapeutic effect and survival of *CDK12* alteration cases. Since only one patient monoallelic loss had CRPC and died, the difference in therapeutic effect depending on the *CDK12* allelic status could not be evaluated. Overall, first-line CRPC therapy was effective in some cases, but second-line CRPC therapy seemed to be ineffective (Table S2). To discuss the therapeutic effect in *CDK12*-altered cases, we need to increase the number of patients.

Several case studies involving *CDK12* alterations have been reported recently (Table S3). Although PSA response rates with ICI have been reported at 11–50% [4–6, 11, 17, 18], the allele status of *CDK12* has not always been reported. Wu et al. explained that *CDK12* biallelic loss increases the number of FTDs, which are genome duplications in the size range of 10 kb–10 Mb, thereby forming a fusion neoantigen and resulting in immunogenicity [4]. Rescigno et al. reported that *CDK12* biallelic loss had more DNA copy breaks and lymphocyte infiltration, but there was no correlation between copy number breaks and tumor-infiltrated lymphocyte density [7]. Furthermore, Schweizer et al. found no correlation between the presence of FTDs and efficacy of ICI [6]. Hence, further investigation is needed to determine whether the fusion neoantigens from FTD caused by *CDK12* biallelic loss is the genuine mechanism for ICI efficacy. Moreover, to establish a therapeutic strategy for *CDK12*-altered prostate cancer, clinical trials based on *CDK12* allelic status and FTD burden are expected.

Our study has several limitations. First, we defined biallelic loss as 2 or more mutations in the sample, but if there are multiple mutations on the same allele, it would not present biallelic loss. Second, FoundationOne^®^ Liquid CDx does not disclose the detailed criterion for variant calling, and CDK12 variants with a low VAF of 0.2% were judged as significant. Because CDK12 mutation is one of the characteristic features of prostate cancer and is not seen in clonal hematopoiesis, we considered that false positives are unlikely. However, the accuracy of variant calling using cfDNA with low tumor content remains controversial. Third, given that we did not perform an epigenome analysis, there might have been cases of biallelic loss due to epigenetic change. Lastly, due to the lack of cases treated with PARP inhibitor or ICI, the therapeutic efficacy was not examined in the Japanese cohort.

In conclusion, we present 12 Japanese cases of *CDK12*-altered prostate cancer from various backgrounds, some of which had an aggressive course, while others achieved long-term survival with existing medical treatment. This case series suggests that a heterogeneity exists in patients with prostate cancer with *CDK12* alterations. Accumulating cases with detailed information will contribute to precision oncology for *CDK12*-altered prostate cancer.

## Supplementary Information

Below is the link to the electronic supplementary material.

Supplementary file 1 Figure S1. Copy number plots of patients with CDK12 alteration excluding KOURO_31-408 shown in Figure 2. Not available for KOURO_liq-11703 and KOURO_liq-11002. Vertical axis shows the row copy number before correction for tumor content. (PDF 9480 KB)

Supplementary file 2 Table S1. Gene list of mutations excluding CDK12 for each case (XLSX 13 KB)

Supplementary file 3 Table S2. Prostate-specific antigen response rate (XLSX 12 KB)

Supplementary file 4 Table S3. CDK12 alteration case series (XLSX 11 KB)

## References

[1] Nguyen B, Mota JM, Nandakumar S (2020). Pan-cancer analysis of CDK12 alterations identifies a subset of prostate cancers with distinct genomic and clinical characteristics. Eur Urol.

[2] Pilarova K, Herudek J, Blazek D (2020). CDK12: cellular functions and therapeutic potential of versatile player in cancer. NAR Cancer.

[3] de Bono J, Mateo J, Fizazi K (2020). Olaparib for metastatic castration-resistant prostate cancer. N Engl J Med.

[4] Wu YM, Cieślik M, Lonigro RJ (2018). Inactivation of CDK12 delineates a distinct immunogenic class of advanced prostate cancer. Cell.

[5] Reimers MA, Yip SM, Zhang L (2020). Clinical outcomes in cyclin-dependent kinase 12 mutant advanced prostate cancer. Eur Urol.

[6] Schweizer MT, Ha G, Gulati R (2020). CDK12 -Mutated prostate cancer: clinical outcomes with standard therapies and immune checkpoint blockade. JCO Precis Oncol.

[7] Rescigno P, Gurel B, Pereira R (2021). Characterizing CDK12-mutated prostate cancers A C. Clin Cancer Res.

[8] Priestley P, Baber J, Lolkema MP (2019). Pan-cancer whole-genome analyses of metastatic solid tumours. Nature.

[9] Sokol ES, Pavlick D, Frampton GM (2019). Pan-cancer analysis of CDK12 loss-of-function alterations and their association with the focal tandem-duplicator phenotype. Oncologist.

[10] Scher HI, Morris MJ, Stadler WM (2016). Trial design and objectives for castration-resistant prostate cancer: Updated recommendations from the prostate cancer clinical trials working group 3. J Clin Oncol.

[11] Chakraborty G, Armenia J, Mazzu YZ (2020). Significance of BRCA2 and RB1 co-loss in aggressive prostate cancer progression. Clin Cancer Res.

[12] Izawa M, Kosaka T, Nakamura K (2021). Pulmonary metastasis secondary to abiraterone-resistant prostate cancer with homozygous deletions of BRCA2: first Japanese case. IJU Case Reports.

[13] Zhou Z, Li M (2021). Evaluation of BRCA1 and BRCA2 as indicators of response to immune checkpoint inhibitors. JAMA Netw Open.

[14] Baba Y, Kosaka T, Kobayashi H (2022). Castration-resistant prostate cancer patient presenting with whole genome doubling with CDK-12 mutation. BMC Med Genomics.

[15] Warner E, Herberts C, Fu S (2021). BRCA2, ATM, and CDK12 defects differentially shape prostate tumor driver genomics and clinical aggression. Clin Cancer Res.

[16] Abida W, Cyrta J, Heller G (2019). Genomic correlates of clinical outcome in advanced prostate cancer. Proc Natl Acad Sci U S A.

[17] Kwon DH, Chou J, Yip SM (2021). Differential treatment outcomes in BRCA1/2-, CDK12-, and ATM-mutated metastatic castration-resistant prostate cancer. Cancer.

[18] Antonarakis ES, Isaacsson Velho P, Fu W (2020). CDK12 -altered prostate cancer: clinical features and therapeutic outcomes to standard systemic therapies, poly (ADP-Ribose) polymerase inhibitors, and PD-1 inhibitors. JCO Precis Oncol.

