# Supplementary figures and images for: Characterizing cyclin-dependent kinase 12(CDK12)-altered aggressive prostate cancer: a twelve-case series
Source: Int J Clin Oncol. 2022 Oct 21;27(12):1867–73. doi: 10.1007/s10147-022-02248-z (PMC9700615; doi:10.1007/s10147-022-02248-z)

KOURO\_20-298

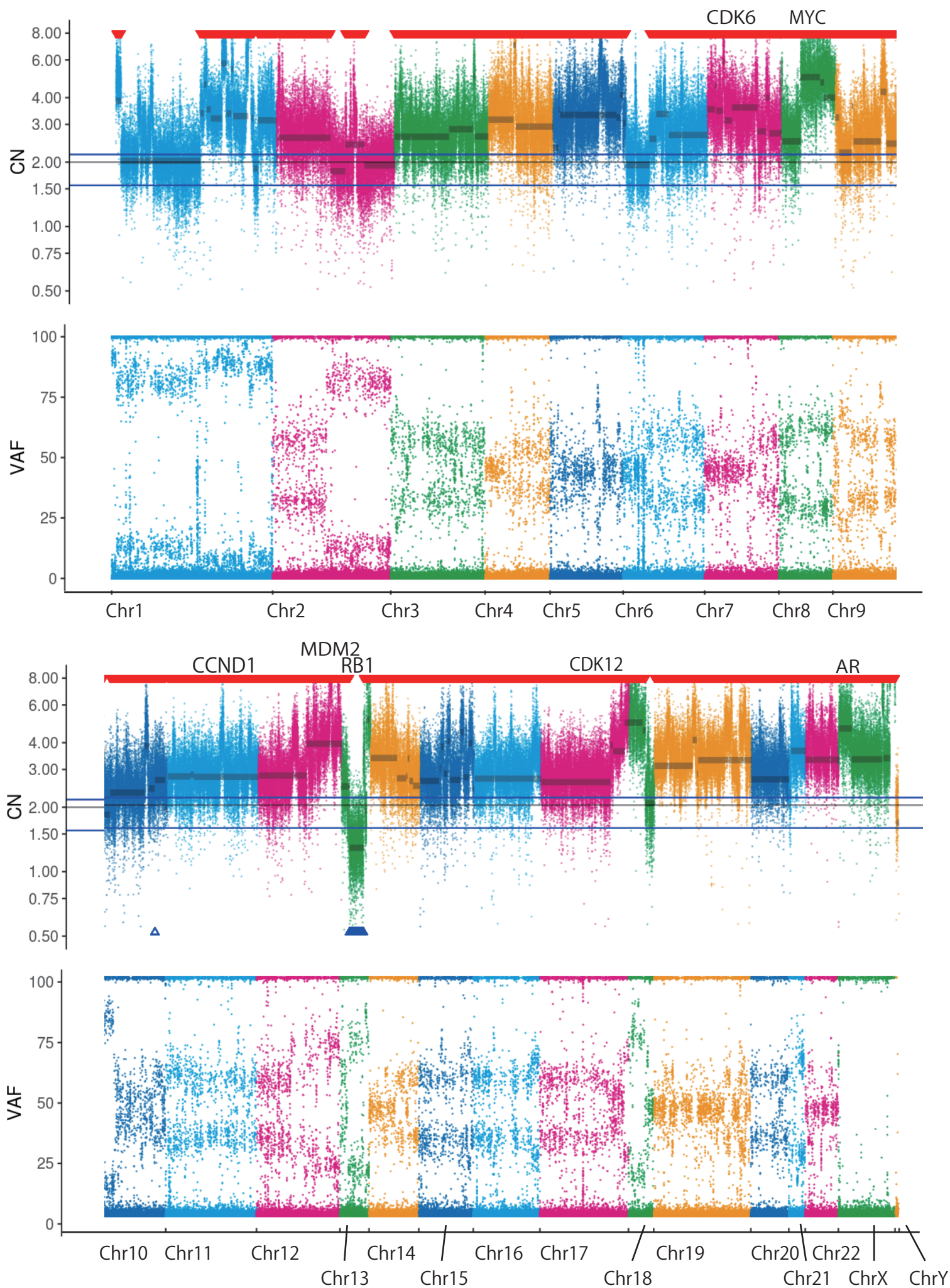

KOURO\_20-331

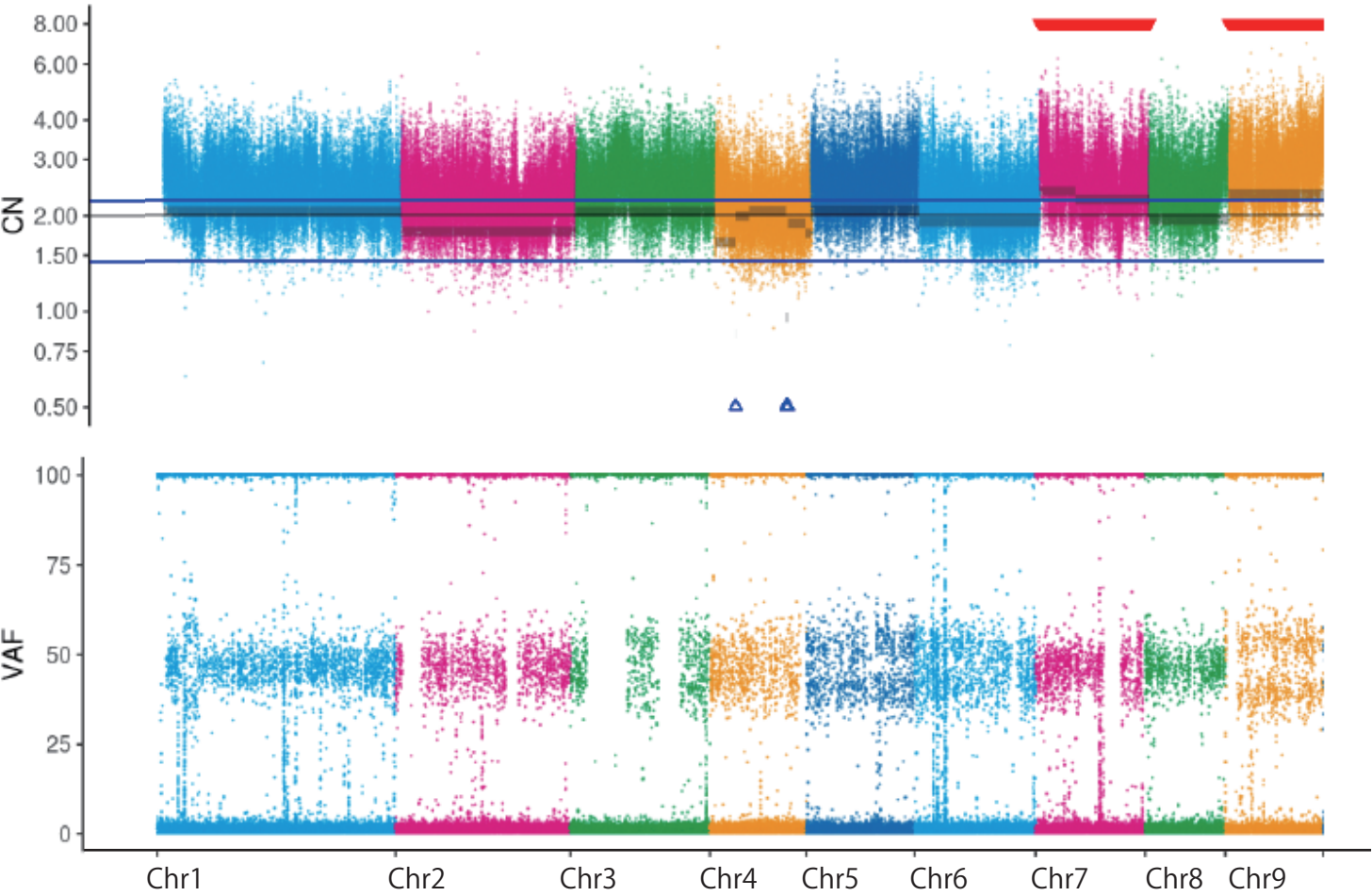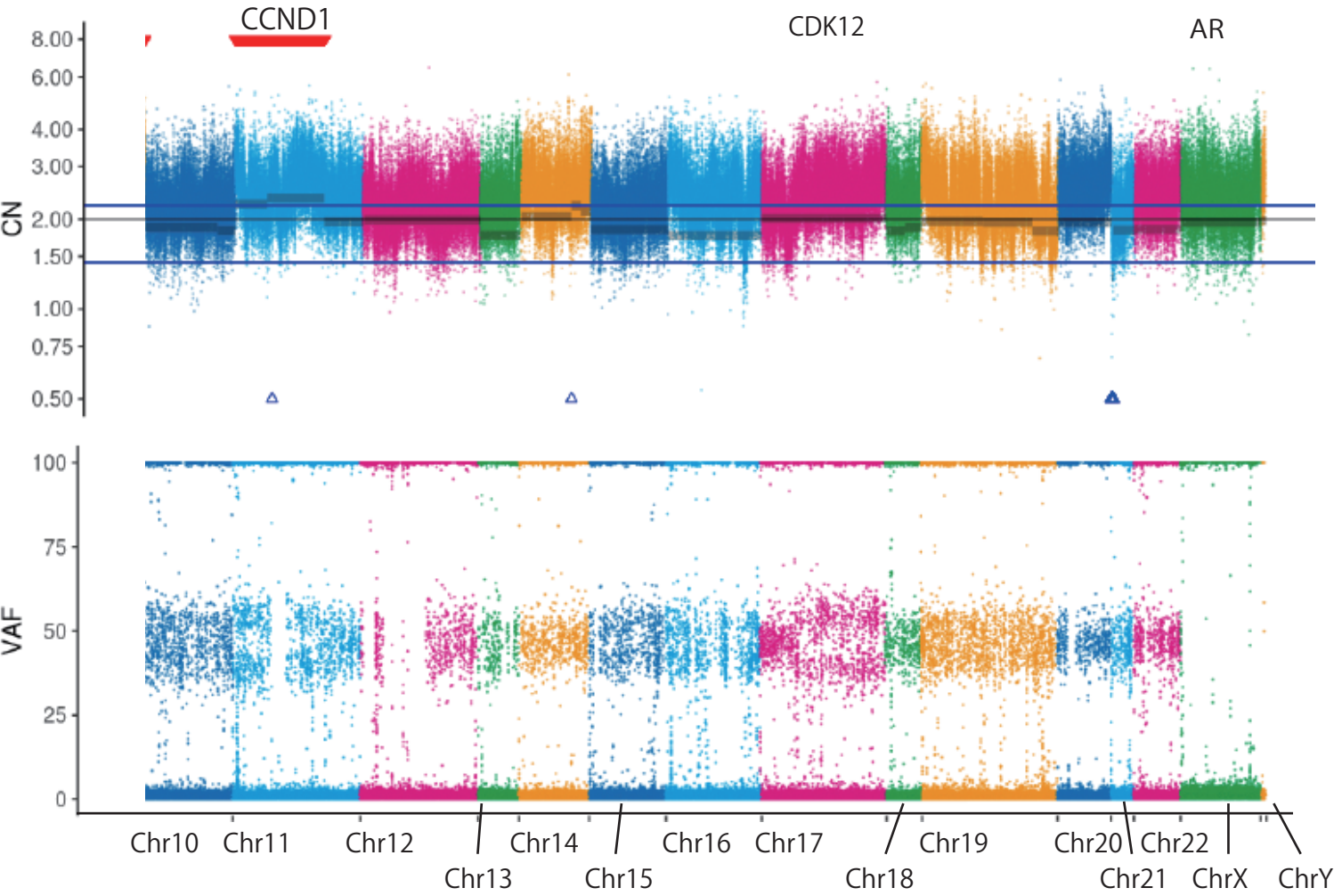

KOURO\_20-334

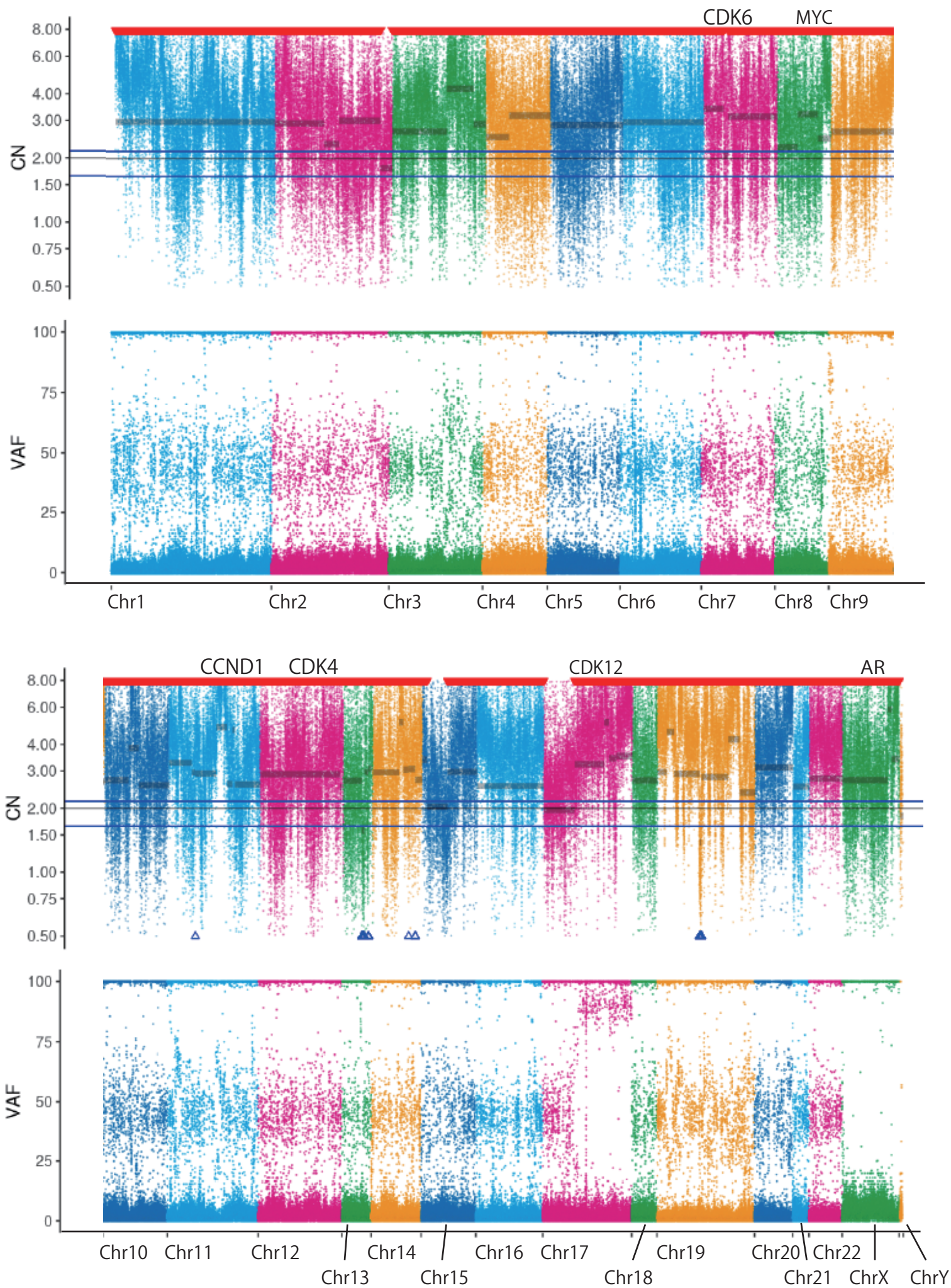

KOURO\_20-346

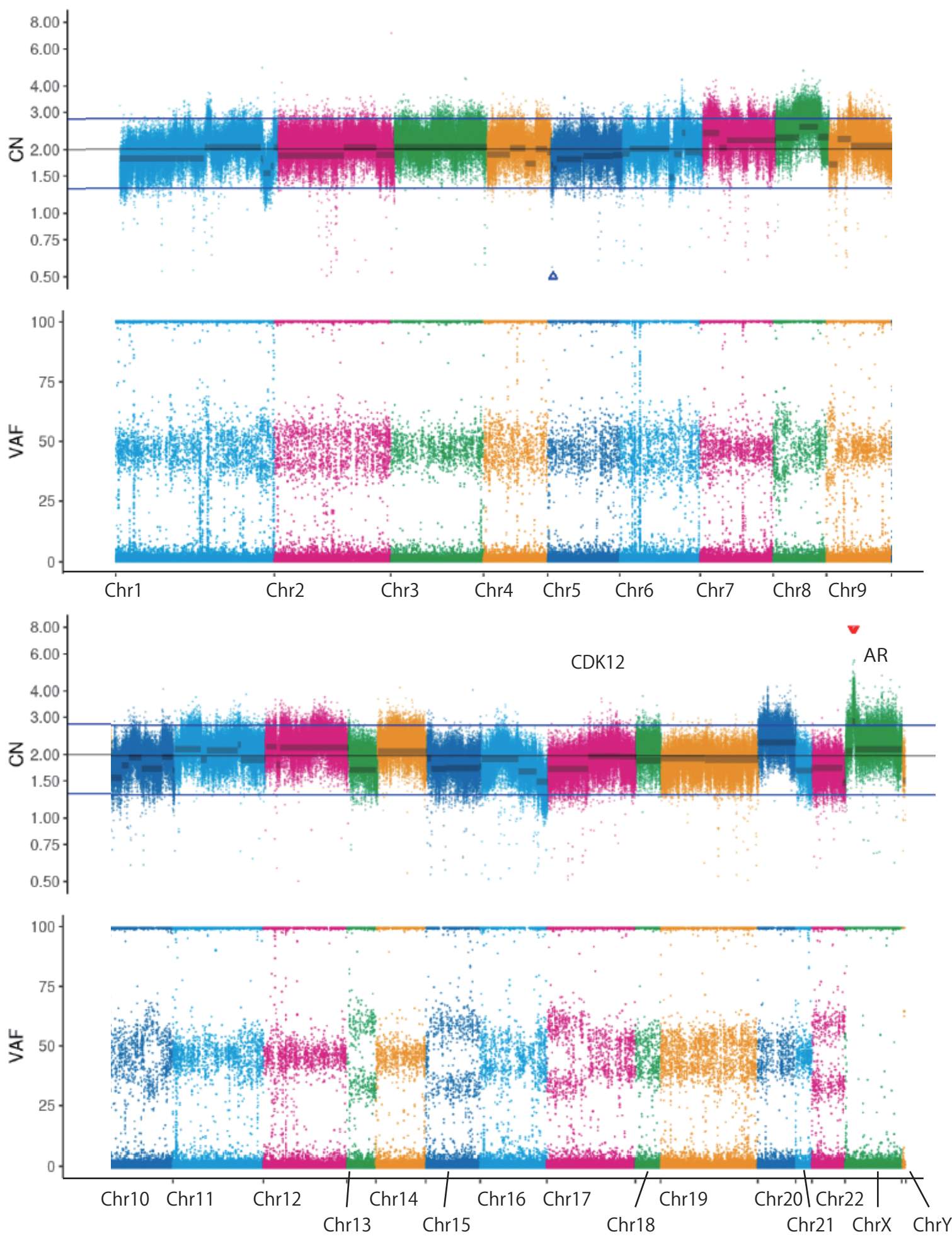

KOURO\_19-598

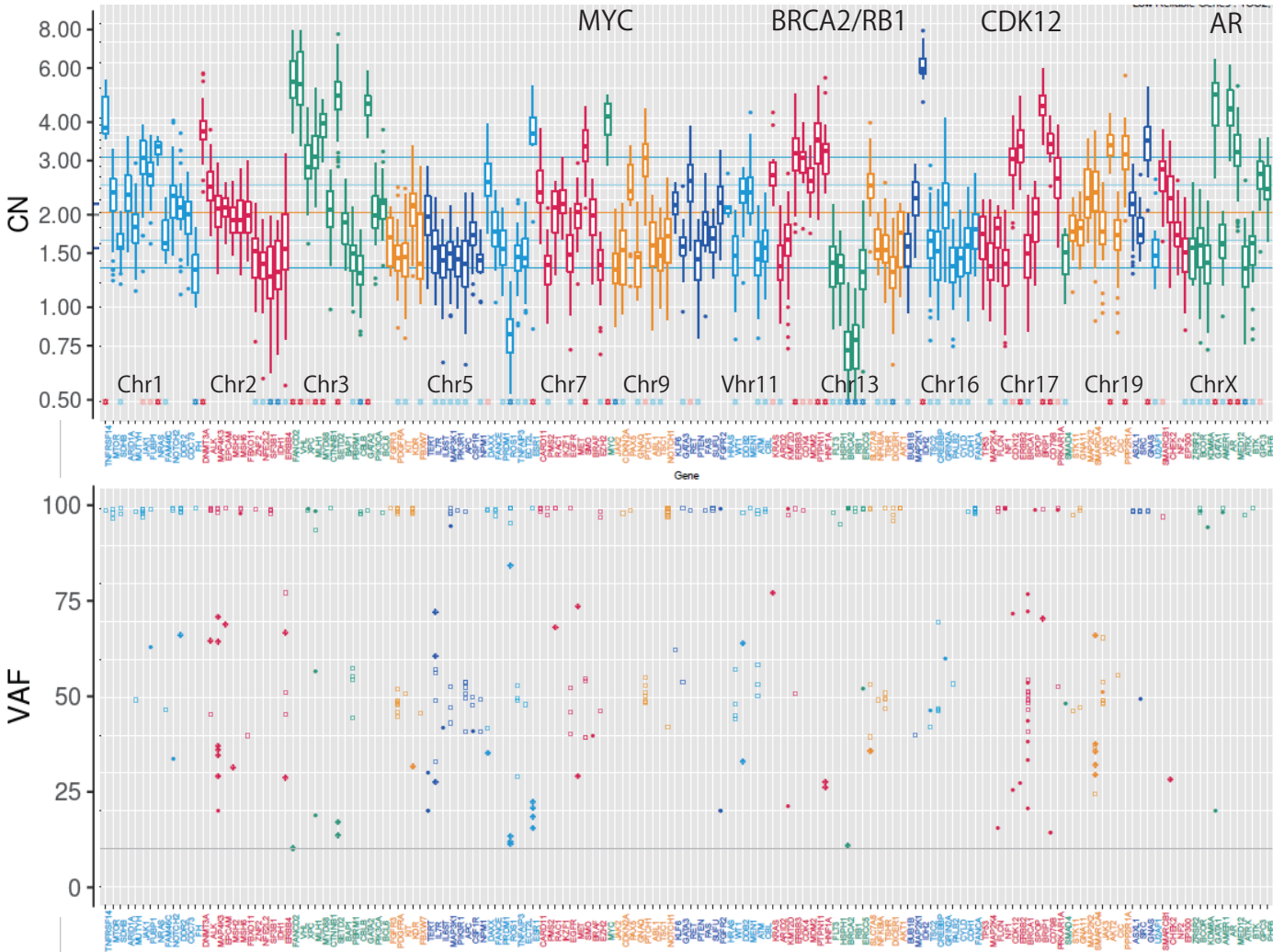

KOURO\_7-15

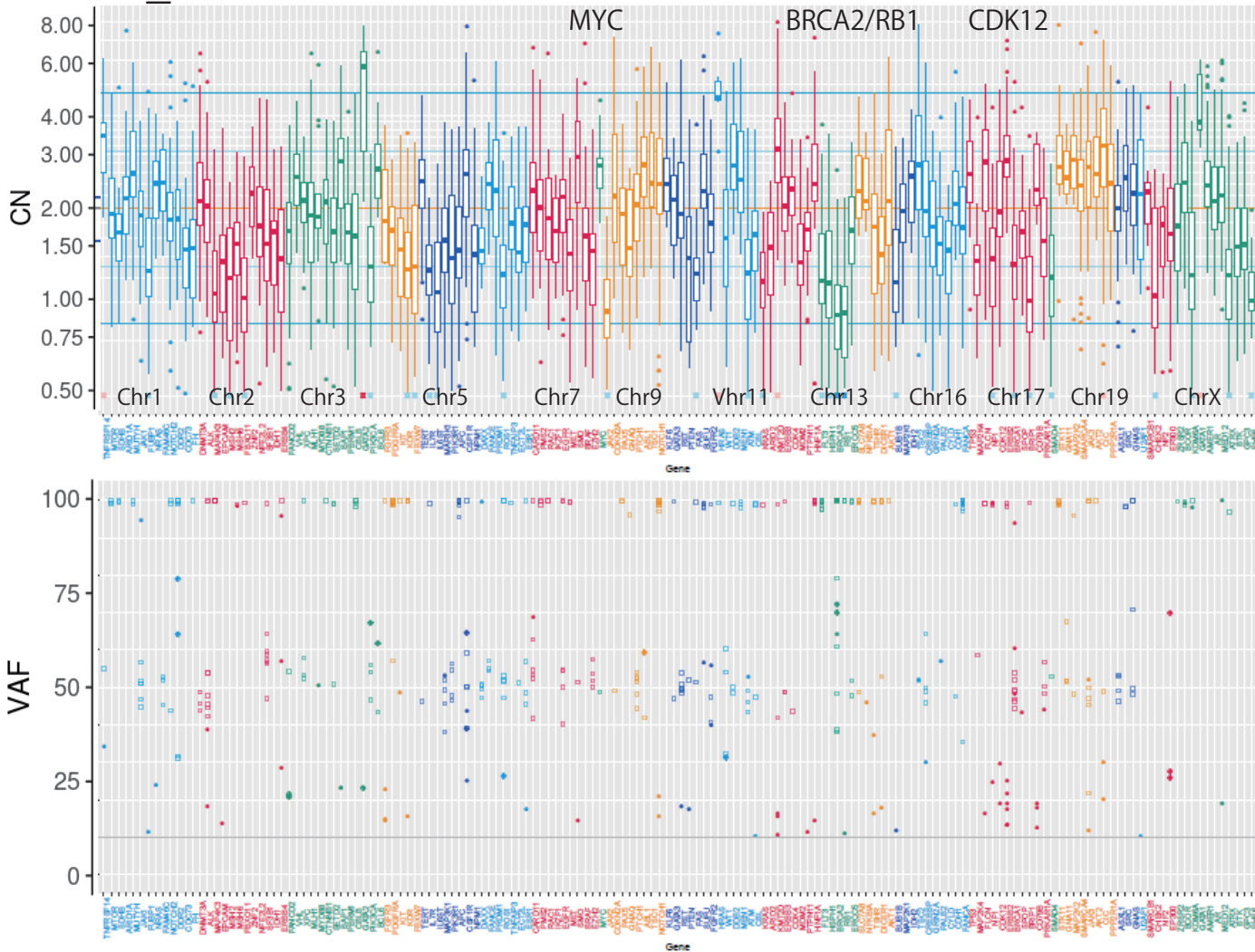

# KOURO\_7-24

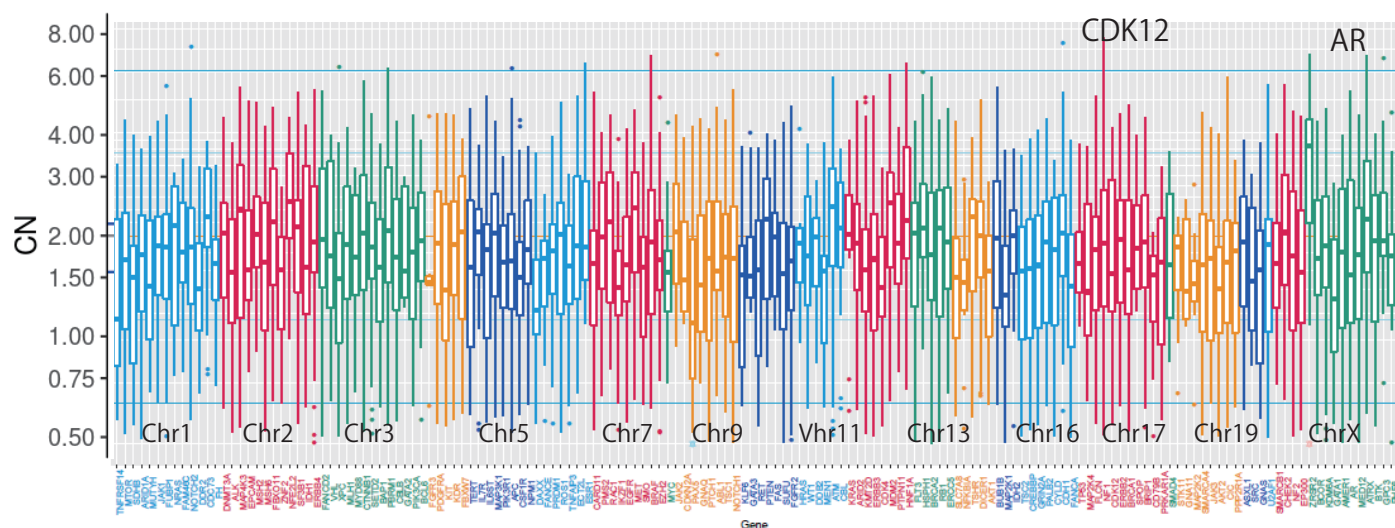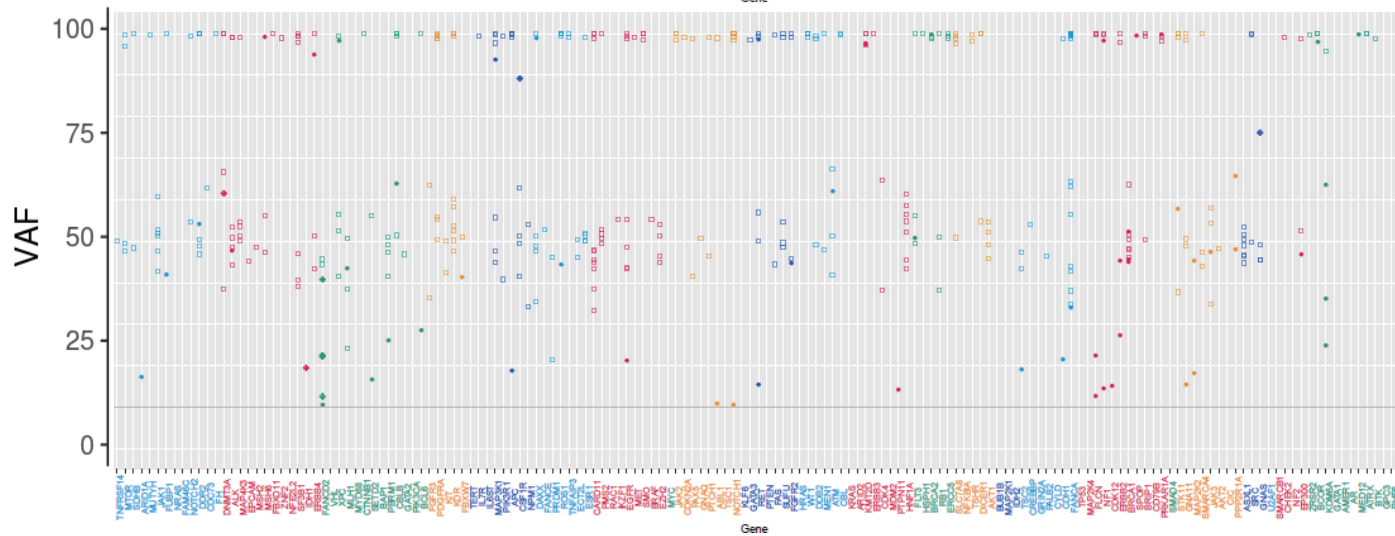

# KOURO\_1-6

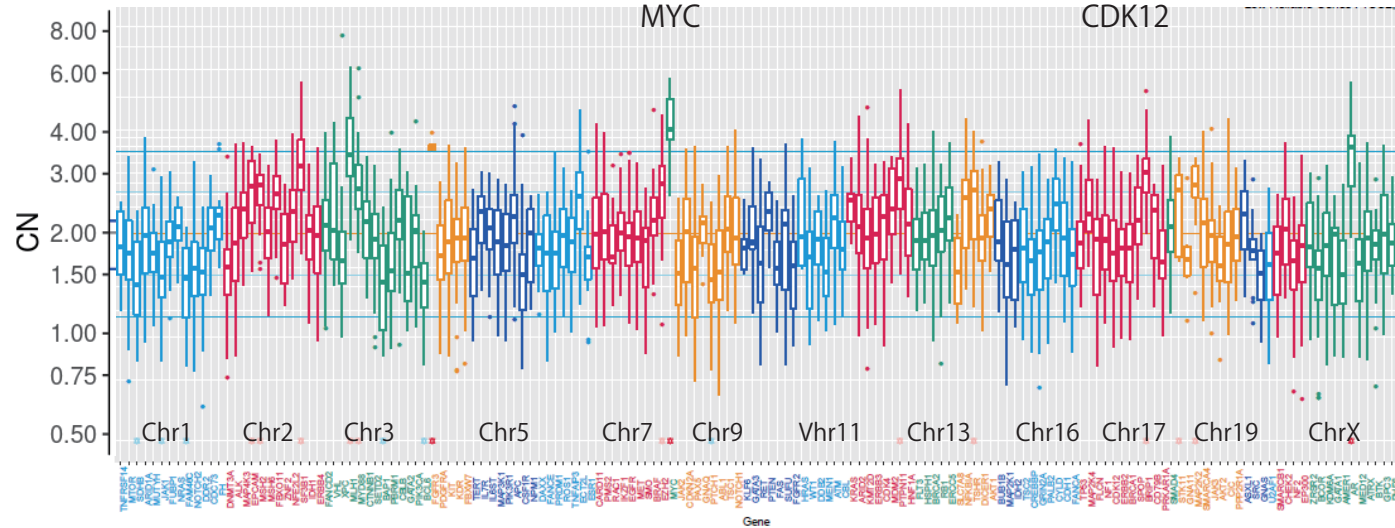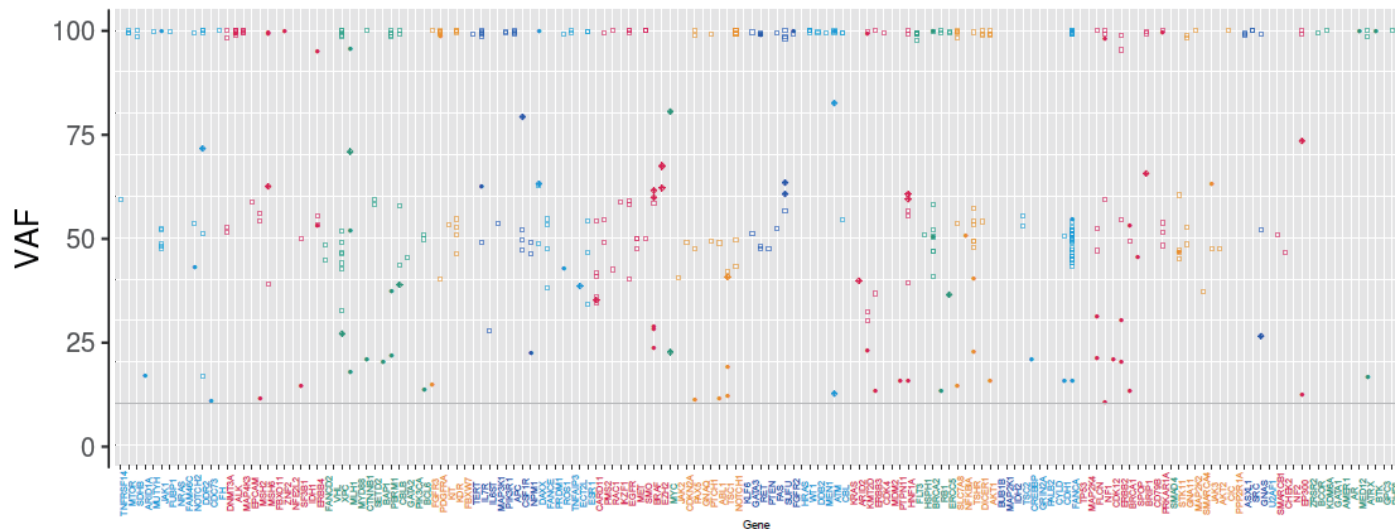

KOURO\_2574

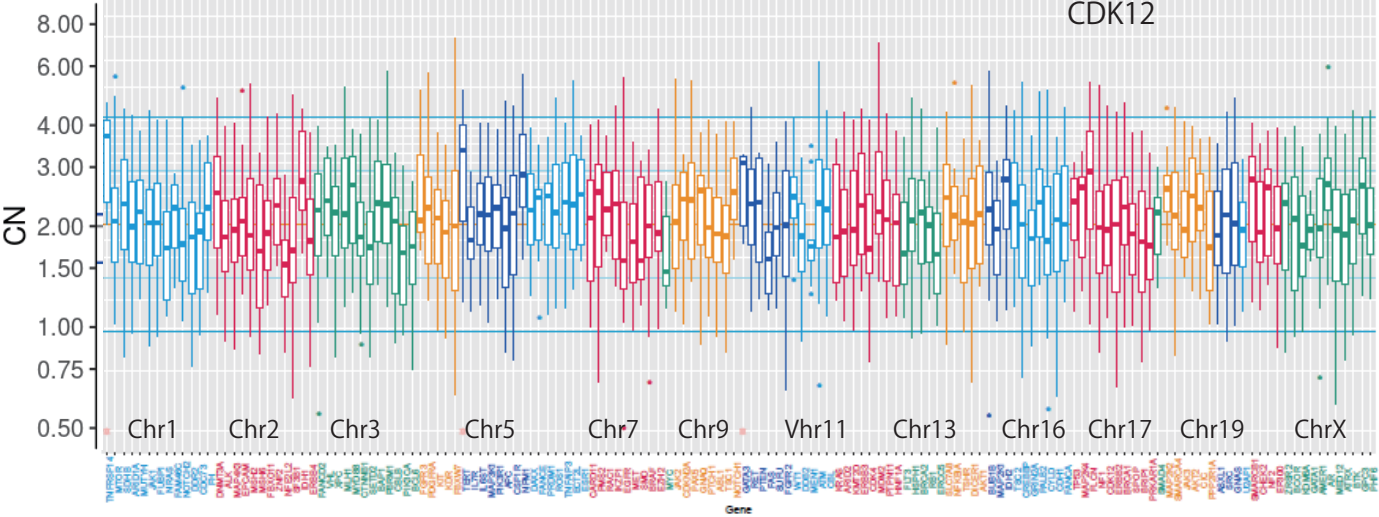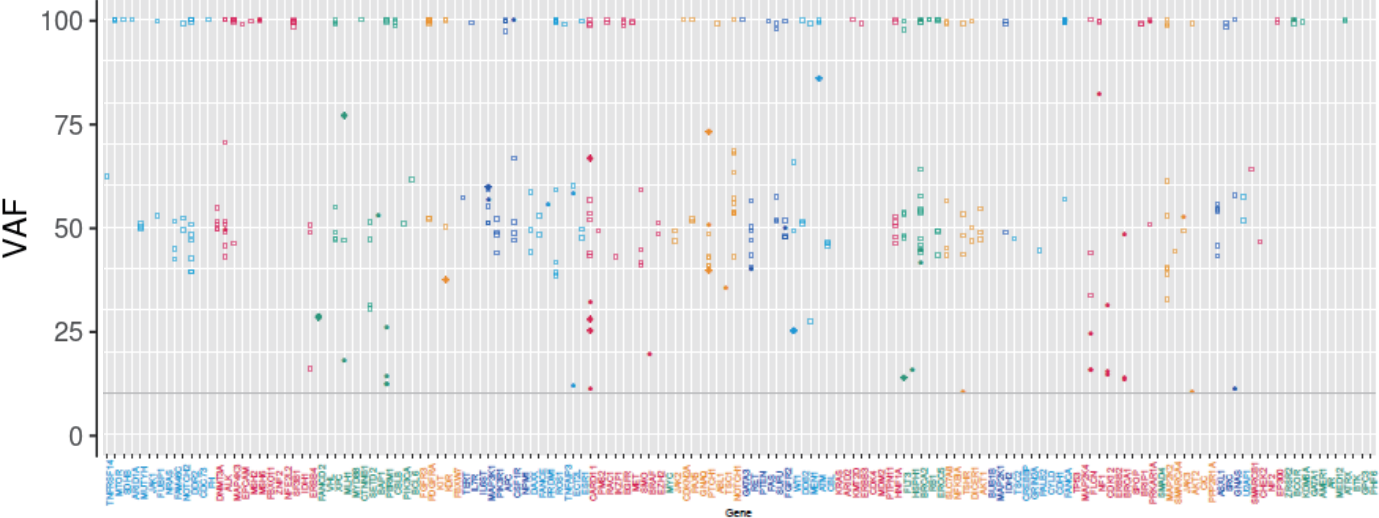

Supplement: Supplementary file 1 — Figure S1. Copy number plots of patients with CDK12 alteration excluding KOURO_31-408 shown in Figure 2. Not available for KOURO_liq-11703 and KOURO_liq-11002. Vertical axis shows the row copy number before correction for tumor content. (PDF 9480 KB) [file 10147_2022_2248_MOESM1_ESM.pdf]
